# Dengue Fever Responses in Dhaka City, Bangladesh: A Cross-Sectional Survey

**DOI:** 10.3389/ijph.2022.1604809

**Published:** 2022-08-30

**Authors:** Md. Mostafizur Rahman, Abu Reza Md. Towfiqul Islam, Saadmaan Jubayer Khan, Kamrun Nahar Tanni, Tuly Roy, Md. Rakibul Islam, Md. Alim Al Raji Rumi, Mohammed Sadman Sakib, Masrur Abdul Quader, Nafee-Ul-Islam Bhuiyan, Musabber Ali Chisty, Farzana Rahman, Edris Alam

**Affiliations:** ^1^ Department of Disaster Management and Resilience, Faculty of Arts and Social Sciences, Bangladesh University of Professionals, Mirpur Cantonment, Dhaka, Bangladesh; ^2^ Department of Disaster Management, Begum Rokeya University, Rangpur, Bangladesh; ^3^ Department of Disaster and Human Security Management, Faculty of Arts and Social Sciences, Bangladesh University of Professionals, Mirpur Cantonment, Dhaka, Bangladesh; ^4^ Institute of Disaster Management and Vulnerability Studies, University of Dhaka, Dhaka, Bangladesh; ^5^ Department of Computer Science and Engineering, Independent University, Bangladesh, Dhaka, Bangladesh; ^6^ Faculty of Resilience, Rabdan Academy, Abu Dhabi, United Arab Emirates; ^7^ Department of Geography and Environmental Studies, University of Chittagong, Chittagong, Bangladesh

**Keywords:** COVID-19, dengue, Dhaka city, KAP, Bangladesh

## Abstract

**Objectives:** This study intends to evaluate the Dhaka city residents’ individual views toward DF.

**Methods:** A cross-sectional survey used google forms for collecting data. Python and RStudio were used for data management and analysis. Kruskal-Wallis or Mann-Whitney U test and logistic regression models were performed, where appropriate.

**Results:** In total 1008 individuals participated in a pre-tested KAP survey. More than 20% reported being affected by DF before the survey, where they rated their current places as being moderately safe (43%). In terms of DF control, 65% had good knowledge, and 68% reported good practice, whereas they demonstrated an overall good attitude. The increased knowledge of individuals could contribute to behavioral changes regarding DF. Female residents demonstrated better DF attitudes (OR: 0.69; *p* < 0.05) and practices (OR: 0.66; *p* < 0.01) compared to male residents. Mixed unit residents had poor KAP levels. Educational attainment can also play an essential role in enhancing the attitude level.

**Conclusion:** Overall, dengue surveillance activities with sufficient campaigns are required for behavioral change in Dhaka city. This information could be integrated into other DF-affected countries’ strategies against dengue outbreaks.

## Introduction

Bangladesh’s capital Dhaka has become a hotspot for dengue fever (DF) and the recently emerged COVID-19 pandemic [1–6]. DF, a neglected tropical disease, has a devastating effect on developing societies, notably in Asia [7]. Dengue fever was originally described in 1780; however, the first dengue outbreak in Bangladesh was reported in 1964 as “Dhaka/Dacca fever” [8]. Following then, dengue cases were intermittently reported in Bangladesh, most notably in Dhaka [8]. Additionally, a rapid increase in dengue cases was noted after 2000, with considerable deaths occurring from the massive number of cases in 2019 [9]. It has been a burden on the country, notably on Dhaka, which was hit hard by a rapid dengue outbreak [1]. In 2019, this highly populated city alone accounted for more than half of the country’s dengue cases [9]. According to studies, a high population density, rapid unplanned urbanization, insufficient dengue monitoring operations, disregard for dengue preventative behavior, and climate change might all contribute to a severe dengue outbreak in the city [10, 11]. Additionally, studies anticipated that this city would be the epicenter of the country’s significant dengue outbreaks [12]. Furthermore, a study asserted that the rapid expansion of the dengue outbreak across the country in 2019 was caused by the large movement of dengue-infected persons (many of whom were predicted to be asymptomatic) from Dhaka to other cities around the country [1]. Dengue incidences are believed to be significantly under-reported in Bangladesh due to the country’s ailing healthcare system [13].

Since the city has already been heavily impacted by the COVID-19 pandemic, citizens are concerned about the pandemic’s impact. Even though dengue and COVID-19 both have fever symptoms and asymptomatic cases, the number of registered dengue cases in the pandemic cannot be neglected [14]. As a result, residents of the city have struggled to contain the disease’s devastating impact.

While there is no effective vaccination for DF, health behaviors such as adhering to authentic advice, maintaining a positive attitude, and following proper practices may pave the way for DF elimination [15]. Individual and community behavioral changes, as well as proper government support for DF, can help minimize the rising dengue incidence in Dhaka, and hence throughout the country. Several studies have already been conducted about DF in Bangladesh [1, 11]. However, understanding residents’ essential knowledge, attitude, and practice (KAP) of DF is vital for effective vector-borne disease control [16].

A recent study examined KAP among university students and found a statistically significant relationship between climatic change and the DF KAP level in Bangladesh [11]. However, to the author’s knowledge, there are few cited works on KAP of dengue infections among closed Dhaka city residents during the COVID-19 pandemic. Additionally, considering the recent increase in dengue mortality inside Dhaka city, it is critical to assess the dengue-associated KAP of city residents in order to develop effective vector control and monitoring measures. This study intended to evaluate the dengue fever responses (though measuring KAP level) among Dhaka city residents. The outcome of this study may give crucial baseline data to international, national, and municipal authorities, as well as non-governmental and social groups regulating the DF.

## Methods

### Research Design and Ethical Issue

The current study is an online cross-sectional survey. As the study was conducted during the COVID-19 pandemic, the easiest way to reach respondents was online. The study was confined to Dhaka, which has one of the highest internet usage rates in the country [17].. This study was a part of an approved research project from the Research Ethics Committee (BUP REC0907/2019) of Bangladesh University of Professionals, Dhaka, Bangladesh. It considered all associated ethical issues. The online questionnaire’s cover page clearly described the objectives and ethical issues of the survey. The online consent of respondents was taken, and they remained anonymous.

**Questionnaire** Prior studies were carefully reviewed to adapt and develop the draft questionnaire considering the Bangladesh perspective [11, 16, 18–21]. Afterward, experts’ opinions and a pilot survey were performed to pre-test the questionnaire. A final self-reported questionnaire in both English and local Bengali versions was developed in Google Forms. It had three parts: socio-demographic information; self-rated status of their current location against dengue outbreak and DF history; and the final KAP sections. Two questions such as “How safe do you think your current place is from dengue?” and “Have you had dengue before?” were asked to measure their current location’s safety rating against DF and individual’s DF experience. Residents’ safety rating was measured through 5-points (very safe = 5, safe = 4, moderately safe = 3, unsafe = 2, and very unsafe = 1) Likert scale. DF experience responses had Yes/No/Maybe options (Yes = 1, Maybe = 0.50, and No = 0). A total of 28 items was in the KAP section. Twelve close-ended items comprised the knowledge section (DF is an infectious disease, it can cause death, common symptoms of the disease, vector type, phenotype of the mosquito, breeding sites of the mosquito, biting time, DF risk during pregnancy, multiple DF infection, and activities to reduce DF outbreak). The score range was 0–1, where the ‘Maybe’ option had a 0.50 score. Eight close-ended items with a 5-point Likert scale (Strongly agree = 5, Agree = 4, Neutral = 3, Disagree = 2, and Strongly disagree = 1) were in the attitude section (responsibility to ensure the free of vector mosquito and the breeding sites, only chemical fogging is not enough to stop DF outbreak, regular DF surveillance activities, community’s commitment to control the outbreak, participation in DF control public activity, and taking immediate treatment). The final practice section had 08 close-ended items (communicating the authority for fogging, using mosquito repellent and coil or mosquito-killing tools, checking the mosquito breeding sites, covering and cleaning the water containers, cleaning the plant pots, visiting a hospital for the treatment and following authentic sources for DF) with the binary answers (Yes/No) and 0–1 score range. The Cronbach’s alpha was also calculated, for the internal consistency, as 0.68, 89, and 0.75 for the knowledge, attitude, and practice section, respectively. Cronbach’s alpha >0.60 indicates the acceptable value whereas >0.80 shows an excellent level [22].

### Data Collection and Analysis

Non-probability sampling technique was applied where a group of university students administered the online questionnaire *via* an online platform such as Facebook or WhatsApp, where appropriate. The survey was carried out from August to December 2020, which is the usual DF period in the country [1] The sample size has been assessed following Yamane’s formula [23]:

$$n=\frac{N}{1+N\left(e^{2}\right)}$$

where *n* = sample size, N = population, e = error tolerance.

According to the new “Population and Housing Census 2022″ report from the Bangladesh Bureau of Statistics, the total population of Dhaka city is more than 10.2 million [24]. Therefore, following this population and 0.05 error tolerance, the required sample size was around 400.

Python (version 2.7; Beaverton, OR 97008, United States) and RStudio (version 1.2.5042; Boston, MA, United States) [25, 26] with 95% CI were used for data management and statistical analyses. Descriptive analyses (frequency, percentage, mean, and standard deviation) were calculated where appropriate. Shapiro-Wilk and Kolmogorov-Smirnov tests were conducted to test the normality of data. Since the data were not normally distributed, non-parametric tests such as Kruskal-Wallis or Mann-Whitney U test were carried out to measure the association of socio-demographic profile with the self-rated status and DF experience among the respondents. Bonferroni correction was considered to adjust the *p-*value in a post hoc test (Dunn’s test)*.* In addition, Spearman’s rank correlation was performed to find out the correlation in the KAP domain. Total knowledge, attitude, and practice score were obtained by summing up all respective item scores. Afterward, responses were categorized into “good” and “poor” levels based on an 80% cut-off point [27]. For instance, 9.6 was calculated as 80% of the total knowledge score [12]. Equal to or higher than 9.5 was considered a ‘good’ knowledge level. Logistic regression analysis was performed where the good and poor level was coded as 1 and 0, respectively. After screening, predictors with only *p* < 0.25 value [28] in univariate analyses were considered in multiple logistic regression analysis.

## Results

### Socio-Demographic Profile

Approximately 1100 participants were approached and 1008 individuals completed the online questionnaire. Thus, the response rate was 91.64%. Table 1 shows that most respondents were at a younger age of 18–25 years (74%). There were more male respondents (53%) than female respondents (47%). Most respondents lived with their families (80%), and a majority of respondents belonged to the Dhaka North City Corporation area (61%). In addition, many respondents were living in high-rise residential units (53%).

**TABLE 1:** Association of socio-demographic information with the self-rating and dengue fever experience (*n* = 1008) (Dhaka, Bangladesh. 2020).

Features	n (%)	Self-rated status of current Place’s safety against DF (Mean ± SD^a^)	DF experience (Mean ± SD^a^)
1. Age (year)		p value = 0.292	p < 0.001
Less than 18	16 (1.59)	3.50 ± 1.32	0.22 ± 0.36
18–25	747 (74.11)	3.17 ± 0.93	0.20 ± 0.40
26–35	180 (17.86)	3.22 ± 0.92	0.25 ± 0.42
36–45	44 (7.34)	3.34 ± 0.99	0.42 ± 0.48
More than 45	21 (2.08)	3.19 ± 0.98	0.52 ± 0.5
2. Gender		p value = 0.222	p value = 0.009
Male	535 (53.08)	3.15 ± 0.99	0.26 ± 0.42
Female	473 (46.92)	3.23 ± 0.87	0.20 ± 0.39
3. Living with Family		p value = 0.033	p value = 0.444
Yes	811 (80.46)	3.21 ± 0.94	0.22 ± 0.41
No	197 (19.54)	3.08 ± 0.94	0.25 ± 0.43
4. Location		p value = 0.912	p value = 0.308
Dhaka South City Corporation	387 (38.39)	3.18 ± 0.93	0.24 ± 0.42
Dhaka North City Corporation	621 (61.61)	3.19 ± 0.95	0.22 ± 0.40
5. Accommodation		p value = 0. 0.009	p value = 0.132
High-Rise^b^	539 (53.47)	3.21 ± 0.92	0.23 ± 0.41
Low-Rise^c^	407 (40.38)	3.23 ± 0.94	0.21 ± 0.40
Mixed	62 (6.15)	2.77 ± 1.03	0.31 ± 0.45
6. Education		p value = 0.170	p value = 0.002
Tertiary (University)	914 (90.67)	3.30 ± 1.16	0.34 ± 0.45
Lower than Tertiary	94 (9.33)	3.18 ± 0.91	0.22 ± 0.40
7. Occupation		p value = 0.014	p value = 0.132
Employed	219 (21.73)	3.31 ± 0.90	0.24 ± 0.42
Unemployed	136 (13.49)	2.99 ± 1.14	0.35 ± 0.46
Students	653 (64.78)	3.19 ± 0.90	0.20 ± 0.39

a SD, Standard deviation.

b High Rise = More than 5-story residential unit.

c Low Rise = 5-story or less than 5-story residential unit.

### Self-Rated Status and Dengue Fever Experience

The majority of the respondents rated moderately safe places (43%) against DF (Table 1), followed by safe (29%), and unsafe (16%) places. Respondents living with their families rated significantly safer places against DF than the respondents who were not with their families. A significant association was also shown in the case of residential units and safety ratings. The high-rise and low-rise building residents rated their places as better than those living in mixed residential units (e.g., containing shops, manfacturing, offices, and residences in the same unit). The employed respondents rated their living places safer than the unemployed respondents.

Around 21% of respondents confirmed their DF experience. Respondents’ age, gender, education level, and present occupation were associated with DF experience. The elder respondents (36–45 years and older) reported significantly more DF experience than the younger (18–25 years). In addition, male respondents significantly experienced more DF than their female counterparts.

### KAP Regarding Dengue Fever

Table 2 shows that the majority of respondents correctly demonstrated their responses regarding the questions about fatal consequences due to DF (93%), common symptoms of DF (88%), female *Aedes* mosquito is responsible for dengue virus transmission (88%), it has a stripe on the body (80%), it can breed both indoors and outdoors (78%), usual biting time is early in the morning, and late evening (76%), a person can be infected multiple times (73%), and possible DF outbreak control activities (keeping the surrounding areas clean and demolishing potential breeding sites) (90%). However, many participants did not know where the vector *Aedes* mosquito bred. They also did not realize that the virus could be transmitted from a pregnant mother to the fetus. In all, 65% of the respondents showed good knowledge based on an 80% cut-off point.

**TABLE 2:** Knowledge, attitude, and practices towards dengue fever (Dhaka, Bangladesh. 2020).

Knowledge						
No	Questions		Correct response [n (%)]		Wrong response [n (%)]	
K1	Do you know dengue is an infectious disease?		648 (64.29)		360 (35.71)	
K2	Do you know dengue fever can cause death?		934 (92.66)		74 (7.34)	
K3	Do you know the common symptoms of dengue infection are rash, headache, high fever, joint pain, muscle pain, nausea?		887 (88.00)		121 (12.00)	
K4	By which type of Aedes mosquito dengue virus is transmitted?		888 (88.10)		120 (11.90)	
K5	Does Aedes mosquito has stripes on the body?		802 (79.56)		206 (20.44)	
K6	Dengue virus can be transmitted through direct contact with an infected person		645 (63.99)		363 (36.01)	
K7	Do you know where Aedes mosquitoes breed?		527 (52.28)		481 (47.72)	
K8	Aedes mosquito breed both indoor and outdoor		790 (78.37)		218 (21.63)	
K9	Aedes mosquito likes to bite early in the morning and late evening		764 (75.79)		244 (24.21)	
K10	Do you know dengue virus can be transmitted from infected pregnant mother to fetus?		545 (54.07)		463 (45.93)	
K11	Do you know one person can be infected with the dengue virus more than once?		740 (73.41)		268 (26.59)	
K12	Do you think dengue fever outbreak can be reduced by keeping you surrounding areas clean and demolishing potential breeding sites?		912 (90.48)		96 (9.52)	
**Attitude**						
**No**	**Statements**	**^a^SA [n(%)]**	**^a^A [n(%)]**	**^a^N [n(%)]**	**^a^DA [n(%)]**	**^a^SDA [n(%)]**
A1	It is my responsibility to make sure there are no Aedes eggs and/or larvae in my house area	483 (47.92)	440 (43.65)	68 (6.75)	13 (1.29)	04 (0.40)
A2	My family members and neighbors should clean Aedes mosquito breeding site like water containers, storage tank, plant pot one to three times a week	558 (55.36)	388 (38.49)	46 (4.56)	11 (1.09)	05 (0.50)
A3	Only chemical fogging by the authority is not enough to prevent dengue infection, authority should also demolish the potential breeding sites	569 (56.45)	369 (36.61)	58 (5.75)	08 (0.79)	04 (0.40)
A4	We should check dengue situation or hotspots around our area regularly	497 (49.31)	410 (40.67)	66 (6.55)	29 (2.88)	06 (0.60)
A5	It is necessary to continue the removal of mosquito breeding sites even during the period when there is no outbreak	468 (46.43)	452 (44.84)	58 (5.75)	18 (1.79)	12 (1.19)
A6	Dengue outbreak in my community can be controlled if every household is committed to remove mosquito breeding sites	494 (49.01)	413 (40.97)	71 (7.04)	23 (2.28)	07 (0.69)
A7	I will take part in a public activity for dengue control or removal of mosquito breeding sites	346 (34.33)	481 (47.72)	162 (16.07)	12 (1.19)	07 (0.69)
A8	If my family member has symptom of dengue fever, i will bring him/her to see a doctor for immediate treatment	581 (57.64)	367 (36.41)	46 (4.56)	10 (0.99)	04 (0.40)
**Practices**						
	**Questions**		**Yes [n(%)]**		**No [n(%)]**	
P1	Do you call municipality authority for fogging?		284 (28.17)		724 (71.83)	
P2	Do you use aerosol and/or liquid mosquito repellent and/or mosquito coil and/or electrical mosquito mat and/or mosquito bed net?		912 (90.48)		96 (9.52)	
P3	Do you properly cover water containers used for water storage?		889 (88.19)		119 (11.81)	
P4	Do you scrub and clean the inner sides of the containers?		850 (84.33)		158 (15.67)	
P5	Do you check for the presence of Aedes eggs and/or larvae inside or outside the house?		543 (53.87)		465 (46.13)	
P6	Do you keep the plant pots clear and drain the extra water?		872 (86.51)		136 (13.49)	
P7	Do you go to hospital for test and treatment when you see the symptoms of dengue?		843 (83.63)		165 (16.37)	
P8	Do you follow the trusted sources of information, such as who or your local and national health authorities?		804 (79.76)		204 (20.24)	

a SA, strongly agree, A = Agree, N=Neutral, DA = Disagree and SDA = strongly disagree.

Individuals agreed that they, their family members, surrounding communities, and appropriate authorities are responsible for cleaning the *Aedes* mosquito’s potential breeding sites and ensuring no *Aedes* mosquito eggs and larvae around their places. They also agreed that these activities should be monitored and maintained regularly, even when the outbreak is not apparent. The individuals also apprehended that they should participate in the public DF control activities. Respondents showed a good attitude (86%) based on an 80% cut-off point.

In total, 68% of respondents demonstrated good practices based on the cut-off point, and 90% of respondents indicated that they used mosquito control or killing appliances and solutions. They also cover (88%) and clean (84%) their water storage containers and, keep plant pots clear (87%), and visit a hospital for DF test and treatment (84%). However, an enormous number of respondents also demonstrated a lack of other salient practices, such as communication with the local authority for fogging and monitoring the potential *Aedes* mosquito breeding sites.

### Association in the KAP Domain

Positive correlations were found in the KAP domain (Table 3). Individuals with good knowledge demonstrated increased odds of having a good attitude (OR: 3.49; 95% CI: 2.43–5.07) and good practice (OR: 2.26; 95% CI: 1.72–2.98) compared to the individuals with poor knowledge. Participants with good attitudes also showed 4.54 times of good practices than the individuals with poor attitudes.

**TABLE 3:** Association in knowledge, attitude, and practices domain (Dhaka, Bangladesh. 2020).

Association	r-value^a^	p value	OR^c^ (95% CI^b^)	p value
Knowledge and Attitude	0.300	<0.001	3.49 (2.43–5.07)	<0.001
Knowledge and Practice	0.204	<0.001	2.26 (1.72–2.98)	<0.001
Attitude and Practice	0.255	<0.001	4.54 (3.14–6.63)	<0.001

a r-value = correlation coefficient.

b OR, odds ratio.

c CI, confidence interval.

### Determinants of KAP Level

Table 4 summarizes the univariate logistic regression analysis results. Living with family, accommodation, and occupation were significant predictors of knowledge. Participants living in the mixed unit were found to have poorer knowledge than the high-rise unit participants. Attitude, age, gender, accommodation, education, occupation, and DF experience were all significant predictors. Male participants showed fewer good attitudes than female participants. Mixed unit residents, those with below tertiary education, unemployed participants, and individuals without DF experience also showed poor attitudes. For practices against DF, gender, living with family, accommodation, and DF experience were determined as significant predictors. Like attitude, poor practices were found in male and mixed unit residents. However, DF experience also contributed to good practices.

**TABLE 4:** Univariate predictors of knowledge, attitude, and practices level towards dengue fever (Dhaka, Bangladesh. 2020).

Characteristics	Knowledge		Attitude		Practice	
	OR^a^ (95%CI^b^)	p value	OR (95%CI)	p value	OR (95%CI)	p value
1. Age (year)						
18–25	1		1		1	
26–35	0.86 (0.61–1.21)	0.377	0.98 (0.62–1.61)	0.939	1.03 (0.73–1.48)	0.846
36–45	0.61 (0.33–1.14)	0.115	0.68 (0.32–1.61)	0.341	1.40 (0.72–2.95)	0.343
Less than 18	0.51 (0.18–1.40)	0.182	0.33 (0.12–1.07)	0.045*	0.78 (0.28–2.31)	0.633
More than 45	1.02 (0.42–2.71)	0.969	0.38 (0.15–1.08)	0.049*	0.62 (0.26–1.55)	0.291
2. Gender						
Female	1		1		1	
Male	0.98 (0.76–1.27)	0.883	0.69 (0.47–0.98)	0.043*	0.66 (0.50–0.87)	0.003**
3. Living with Family						
Yes	1		1		1	
No	0.71 (0.51–0.97)	0.033*	0.69 (0.46–1.07)	0.090	0.67 (0.48–0.92)	0.014*
4. Location						
Dhaka North City Corporation	1		1		1	
Dhaka South City Corporation	0.78 (0.59–1.01)	0.056	1.04 (0.72–1.51)	0.832	0.92 (0.70–1.21)	0.564
5. Accommodation						
High-Rise	1		1		1	
Low-Rise	0.79 (0.60–1.03)	0.087	1.10 (0.75–1.63)	0.626	1.28 (0.97–1.70)	0.083
Mixed	0.38 (0.22–0.64)	0.000***	0.35 (0.19–0.64)	0.000***	0.56 (0.33–0.95)	0.033*
6. Education						
Tertiary (University)	1		1		1	
Lower than Tertiary	0.67 (0.44–1.04)	0.073	0.37 (0.23–0.62)	0.000***	0.85 (0.54–1.33)	0.463
7. Occupation						
Students	1		1		1	
Employed	0.90 (0.66–1.25)	0.546	0.84 (0.53–1.34)	0.449	1.26 (0.90–1.78)	0.174
Unemployed	0.67 (0.46–0.98)	0.040*	0.33 (0.21–0.52)	0.000***	0.85 (0.58–1.25)	0.402
8. DF Experience						
No	1		1		1	
Maybe	1.09 (0.51–2.46)	0.829	0.73 (0.29–2.20)	0.527	1.39 (0.62–3.55)	0.451
Yes	1.02 (0.74–1.40)	0.920	0.66 (0.44–1.00)	0.043*	0.63 (0.46–0.87)	0.005**

*p < 0.05; **p < 0.01; ***p < 0.001.

a OR, Odds Ratio.

b CI, confidence interval.

Multiple logistic regression analysis shows that only residential units remained a significant predictor of knowledge (Table 5). Gender, accommodation, education, and occupation remained significant predictors of attitude. In the case of practice, gender, accommodation, and DF experience were found as significant predictors.

**TABLE 5:** Multiple logistic analysis to identify the predictors of knowledge, attitude, and practices level towards dengue fever (Dhaka, Bangladesh. 2020).

Characteristics	Knowledge		Attitude		Practice	
	aOR^a^ (95%CI^b^)	p value	aOR (95%CI)	p value	aOR (95%CI)	p value
1. Age (year)						
18–25						
26–35	0.97 (0.62–1.54)	0.916	1.67 (0.92–3.09)	0.938		
36–45	0.70 (0.35–1.41)	0.309	1.19 (0.50–3.10)	0.702		
Less than 18	0.68 (0.24–1.98)	0.474	0.69 (0.22–2.46)	0.542		
More than 45	1.27 (0.48–3.66)	0.634	0.77 (0.26–2.52)	0.654		
2. Gender						
Female						
Male			0.68 (0.46–1.00)	0.049*	0.63 (0.47–0.83)	0.001**
3. Living with Family						
Yes						
No	0.73 (0.53–1.01)	0.055	0.81 (0.53–1.28)	0.363	0.73 (0.53–1.02)	0.068
4. Location						
Dhaka North City Corporation						
Dhaka South City Corporation	0.82 (0.63–1.24)	0.170				
5. Accommodation						
High-Rise						
Low-Rise	0.81 (0.61–1.06)	0.126	1.16 (0.78–1.73)	0.458	1.33 (1.00–1.78)	0.048*
Mixed	0.42 (0.24–0.72)	0.002**	0.42 (0.23–0.80)	0.007**	0.56 (0.32–0.96)	0.035*
6. Education						
Tertiary (University)						
Lower than Tertiary	0.78 (0.49–1.24)	0.294	0.47 (0.28–0.83)	0.007**		
7. Occupation						
Students						
Employed	0.95 (0.61–1.47)	0.804	0.74 (0.42–1.37)	0.331	1.36 (0.96–1.93)	0.086
Unemployed	0.81 (0.52–1.28)	0.370	0.35 (0.20–0.60)	0.000***	0.99 (0.66–1.48)	0.947
8. DF Experience						
No						
Maybe	1.09 (0.51–2.46)	0.829	1.37 (0.50–4.52)	0.565	1.70 (0.74–4.43)	0.236
Yes	1.02 (0.74–1.40)	0.920	0.82 (0.54–1.28)	0.378	0.67 (0.49–0.93)	0.016*

*p < 0.05; **p < 0.01; ***p < 0.001.

a aOR, adjusted odds ratio.

b CI, confidence interval.

## Discussion

Our study shows the findings of the KAP study on DF among Dhaka city residents. Respondents might be knowledgeable and aware of DF due to the online survey pattern. Overall attitude level demonstrated by the study population was good. However, a study carried out in Malaysia reported good knowledge and practices, but poor attitudes towards DF [29]. Regular awareness campaigns, available health facilities, and intensified educational activities could be responsible for the different knowledge, attitudes, and practices.

This study revealed that the study population was concerned about their safety against DF, where numerous respondents had already experienced this disease. This study was conducted when COVID-19 became a major safety concern. Many people were affected and concerned about their safety due to this infectious disease resembling DF. Besides, COVID-19 also has fever-like symptoms, like DF. The overall situation might lead to the respondents’ perception of their safety even though the question was only about DF. The previous DF experiences among individuals again confirm many DF cases in Bangladesh [9]. However, there might also be a bulk number of asymptomatic DF cases that residents or the authorities could not track without testing. We also found that the elder group was more infected than the younger group. Despite the historical prevalence of DF among the children and younger groups in Asia, some studies also recorded the DF prevalence among the elder group [30, 31]. Older people might have greater mortality and morbidity for DF due to age-related immune function [30, 31]. The elderly have likely experienced more dengue outbreaks than the younger generation. This study also revealed that the prevalence of DF among men than the women who responded resembles the prior studies conducted among Asian people [10, 13]. Male groups in Bangladesh have also been more affected by another infectious disease, COVID-19, than their female counterpart [32], which might have biological, behavioral, and cultural responsible factors [33]. In certain circumstances, men exhibit less safe attitudes and practices than women due to gender psychology [34]. Another possible factor is masculinity, which is one of the significant indicators of men’s safety behavior [35]. On the other hand, this study also revealed that the unemployed group experienced more DF than the employed and students. This result likewise supports other studies conducted among Chinese nationals [30]. The mixed unit’s participants were less confident in their location’s safety against DF due to the variability and congested dwellings. People from different backgrounds conducting several business activities without upholding robust DF guidelines might be the reason behind this unsafe rating.

Since this study was conducted during the COVID-19 pandemic, it should be noteworthy that the respondents might face difficulty differentiating the fever symptoms, which are also common for COVID-19. In addition, the current study found that the residents were deemed to require basic DF knowledge. Few participants correctly recognized the dengue risk during pregnancy. The inadequate knowledge regarding dengue risk during pregnancy was also identified in Malaysia [16]. Prior studies already reported this risk [16, 36, 37]. Furthermore, the majority of participants failed to identify the breeding sites for Aedes mosquito accurately, and multiple chances of infection, leading to the high risk of severe DF suggested in a previous study [38].

The study population agreed that fogging alone is not enough to control the dengue outbreak; instead, it requires a holistic approach where individual households, communities, and authorities could work together to remove mosquito breeding sites. It was also suggested by previous studies [39] but contradictory to the findings of other studies [16, 40], where communities mostly agreed on chemical fogging instead to remove mosquito breeding sites. Chemical fogging can be one way to control DF outbreak, with pros and cons to using it [41]. However, few respondents called their municipality for fogging, and many did not monitor the mosquito breeding sites. It revealed the gap between this community and the municipal authority, which was prominent in a recent COVID-19 study among Bangladeshi people [6]. In addition, people might believe that authority would do the fogging when required. Regular monitoring of mosquito breeding sites is required with proactive measures for effective DF control [16]. Many of the study population used adequate measures to kill the mosquitoes. However, most did not monitor mosquito eggs and larvae at home and outside. Inadequate practices to prevent mosquito breeding have indicated the knowledge gap. It also corresponds to another study in Vietnam [18].

Dengue mortality can be reduced through early detection based on patient information [19]. Suppose the health workers and organizations working for DF control have fast-track information such as the socio-demographic condition of individuals as associating factors with the DF responses. In that case, they can intervene and facilitate the effective DF control effort. We found that a better socio-demographic condition could ensure better knowledge, attitude, and practices towards DF. It also corresponds to other studies [16, 42]. The residents living in high-rise units demonstrated better dengue knowledge, attitude, and practice than the mixed unit residents. It shows the high dengue risk among the mixed unit residents. DF can be prominent in this area without effective DF control efforts. All residential units should form a committee to conduct regular DF surveillance activities along with the central and local authorities. This study also revealed that unemployed people have poor knowledge and poor attitude. It also supports the previous studies [16, 43], which argued the involvement of employed people in their workplace’s different DF control programs. Our findings revealed that people with higher education could have a better attitude.

A holistic approach must be considered following the WHO dengue control strategies [44]. The authority’s strategy should ensure proper knowledge dissemination constructive attitude and preventive practices about DF. Along with frequent Aedes mosquito surveillance, governments must organize campaigns, social mobilization, and communication to educate and train citizens on DF management. Online initiatives employing web-based and mobile applications may be useful in the COVID-19 pandemic. Television and social media may also provide community education programs such as short films, case studies, and early warnings on DF outbreak control techniques. Social media has become an increasingly important information source for the general people in Bangladesh [11]. However, literacy and cognitive understanding must be considered in designing and implementing all these strategies [45]. The health and disaster management authorities could also incorporate pre and post-dengue outbreak-related information through these platforms. The authorities must comprehensively equip and train their staff and relevant stakeholders to combat this infectious disease [46]. One study also suggested the substantial interaction between researchers, industries, and communities to formulate effective dengue control strategies [47].

The findings of this research have focused on DF in Dhaka city. However, it can also be applied to other dengue risk areas. Authorities and communities worldwide are concerned and busy from the pandemic. A study found the impact of the COVID-19 pandemic on the vector control program in India [48], which addressed the increased dengue risk due to the lockdown. Thus, it has become urgent to examine the KAP level regarding DF during the pandemic. Our KAP model has indicated that the increased individuals’ knowledge could contribute to behavioral changes such as attitudes and practices regarding DF. It has also determined the associated predictors of KAP level. This information could be integrated into other DF-affected countries’ strategies to control the dengue outbreak.

### Limitations

The current study has some limitations which should be noticed when interpreting the findings. First, it followed non-probability sampling techniques through a self-reported online questionnaire survey during the COVID-19 pandemic. Thus, this survey might not be completely free of some biases. For example, respondents might consider socially acceptable responses even with the anonymized survey pattern. They also required internet access to participate in the survey. Most of them reported a tertiary level of educational attainment. They might also experience DF around their locale since Dhaka city has become an epicenter of DF. For these, the respondents demonstrated good knowledge, attitude, and practices. Second, it only considered Dhaka–the capital city of Bangladesh and the worst affected by DF, where the study population might not reflect the larger population of Bangladesh. Third, the findings were based on a self-reported online survey without further validating the responses. For instance, respondents could report that they were infected by DF; however, it was not validated from their medical records. The authors could not follow up with medical records due to ethical issues and the online survey pattern of the study. Considering all the limitations, this exploratory study could still provide vital information for the Bangladeshi authorities. In addition the findings of this study can assist the other affected communities in integrating into their DF control efforts, particularly during the pandemic period.

### Conclusion

Our study demonstrates necessary information for Bangladesh’s national and local authorities to prepare strategic solutions against the prevalent DF while the ongoing COVID-19 pandemic poses significant challenges. Since all the nations of the world are concerned with pandemic control, appropriate authorities, stakeholders, and the community could ensure effective DF control. Knowledge, attitude, and practices are integral to mitigating DF risk. Vertical and horizontal holistic approaches are required to control this disease. Authorities working on infectious disease control need to strengthen their dengue surveillance activities with other campaigns such as the COVID-19 control program. Following community norms and incorporating adequate traditional knowledge, established national regulations, and international standards with proper authority could significantly reduce DF risk even in a pandemic. The findings urge the required effective knowledge distribution, motivation of the community to a positive attitude, and regular monitoring of the preventive practices to ensure DF control. It also found that the DF control could be possible through enhancing the quality of living standard of the population. Thus, a multi-disciplinary approach is required to change the behavior of residents. It can be attained by having various stakeholders come together and integrate their efforts to combat a common cause. To fight dengue successfully without harm to the aquatic environment, coupling vector control tools will depend primarily on residents’ success and community practices to lessen vector breeding areas.
